# Author Correction: Structure of ATP synthase under strain during catalysis

**DOI:** 10.1038/s41467-022-30544-9

**Published:** 2022-05-17

**Authors:** Hui Guo, John L. Rubinstein

**Affiliations:** 1 Molecular Medicine Program, The Hospital for Sick Children, Toronto, Ontario Canada; 2 Department of Medical Biophysics, The University of Toronto, Toronto, Ontario Canada; 3 Department of Biochemistry, The University of Toronto, Toronto, Ontario Canada

**Keywords:** Molecular conformation, Bioenergetics, Cryoelectron microscopy

Correction to: *Nature Communications* 10.1038/s41467-022-29893-2, published online 25 April 2022.

In the original version of this article, the Data availability section contained typographical errors within the accession codes to Electron Microscopy Database (EMDB) entries. The codes and associated hyperlinks have been corrected in the PDF and HTML versions of the Article.

