# Supplementary material for: Structure of ATP synthase under strain during catalysis
Source: Nat Commun. 2022 Apr 25;13:2232. doi: 10.1038/s41467-022-29893-2 (PMC9038767; doi:10.1038/s41467-022-29893-2)
Supplement: Supplementary file 1 — Supplementary Information [file 41467_2022_29893_MOESM1_ESM.pdf]

SUPPLEMENTARY INFORMATION

Structure of ATP synthase under strain during catalysis

Hui Guo[1,2] and John L. Rubinstein[1,2,3]

1. Molecular Medicine Program, The Hospital for Sick Children, Toronto, Ontario, Canada
2. Department of Medical Biophysics, The University of Toronto, Ontario, Canada
3. Department of Biochemistry, The University of Toronto, Ontario, Canada

* Correspondence: john.rubinstein@utoronto.ca

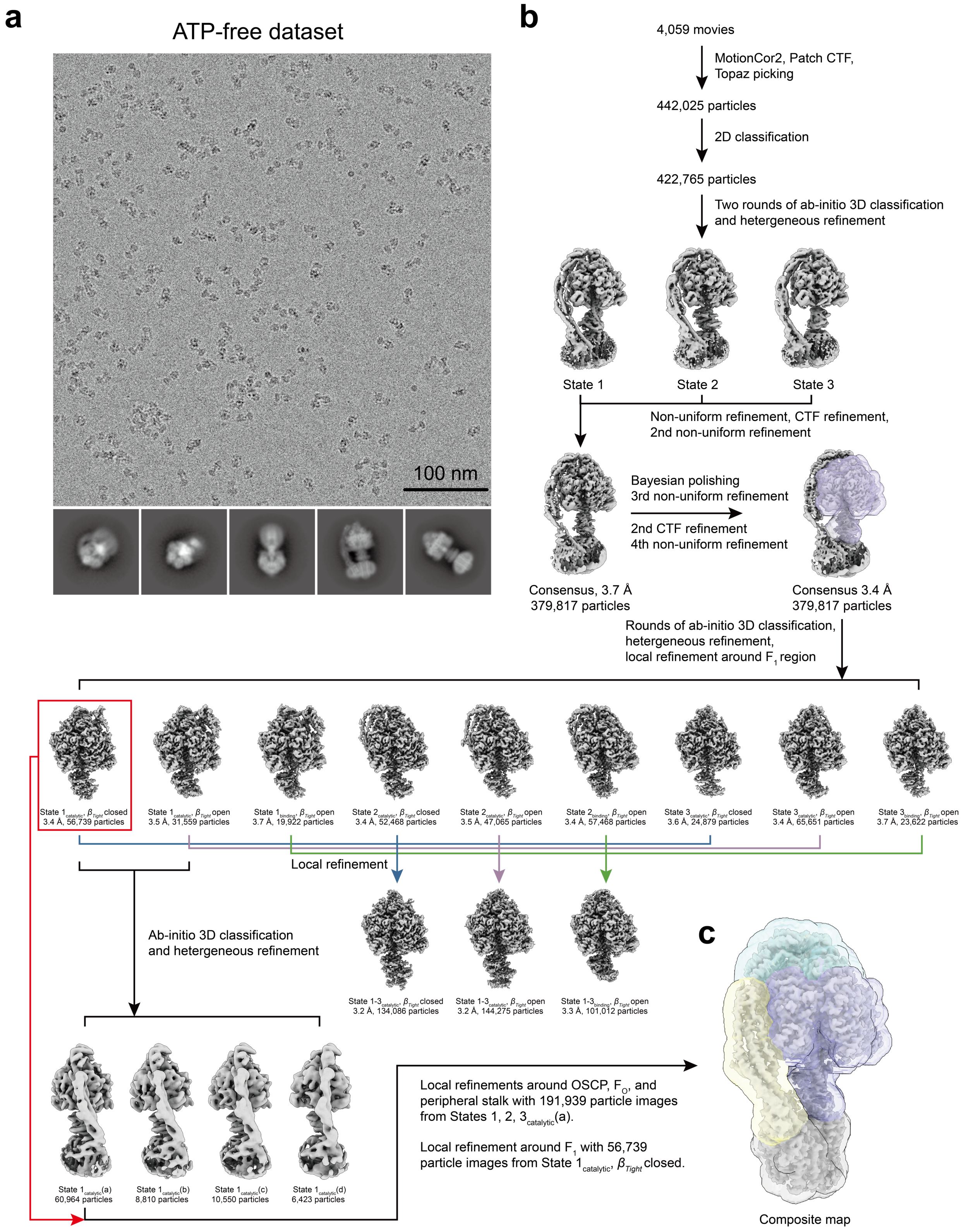

Supplementary Figure 1. CryoEM of the ATP-free dataset. A, Example micrograph (1 out of 4059) and class average images (5 out of 73). **B,** CryoEM data analysis workflow. **C,** Masks used for focused refinement.

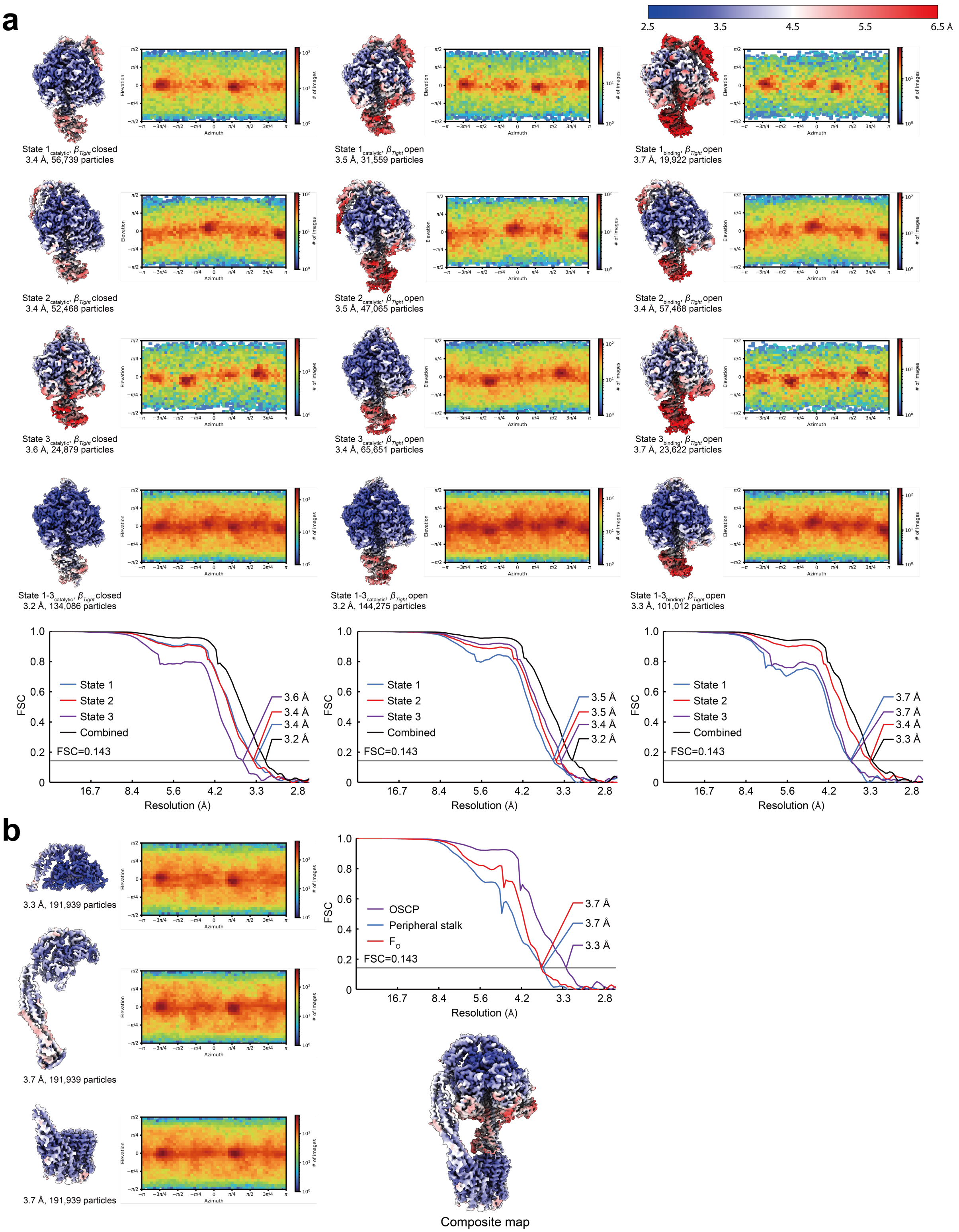

Supplementary Figure 2. CryoEM map validation for ATP-free dataset. A, Local resolution maps, orientation distribution plots, and gold standard Fourier Shell Correlation curves (with masking correction) for the different conformations observed. **B,** Local resolution maps, orientation distribution plots, and gold standard Fourier Shell Correlation curves (with masking correction) for focused refinement.

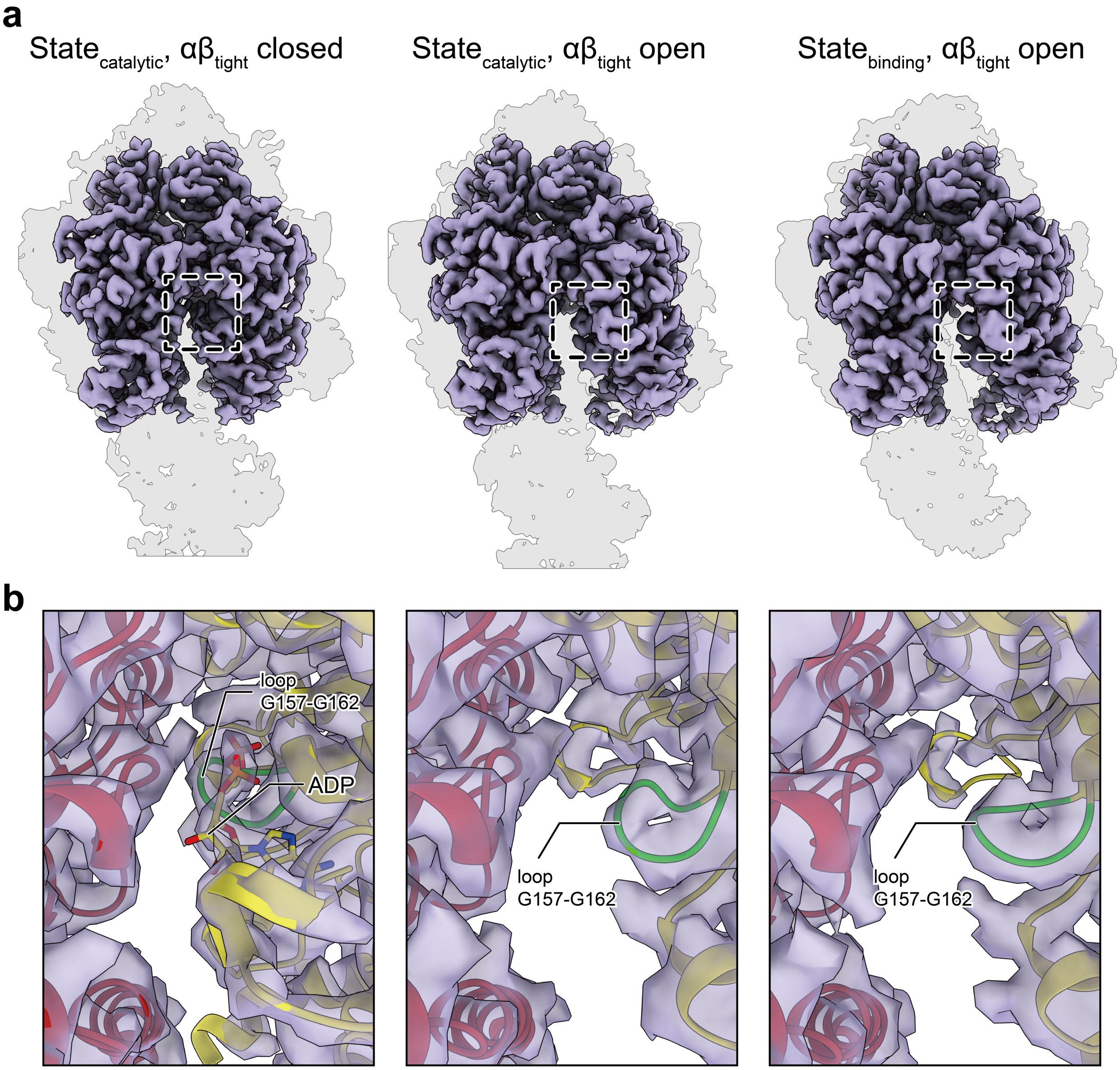

Supplementary Figure 3: Nucleotide content of the αβ$_{tight}$ site in the absence of free ATP. **A,** The αβ$_{tight}$ site shows a closed (*left*) or open *(middle*) conformation in the catalytic dwell structure, and an open conformation (*right*) in the binding dwell structure. **B,** Density for nucleotide in the αβ$_{tight}$ site is apparent only for catalytic dwell structure in the closed conformation (*left*).

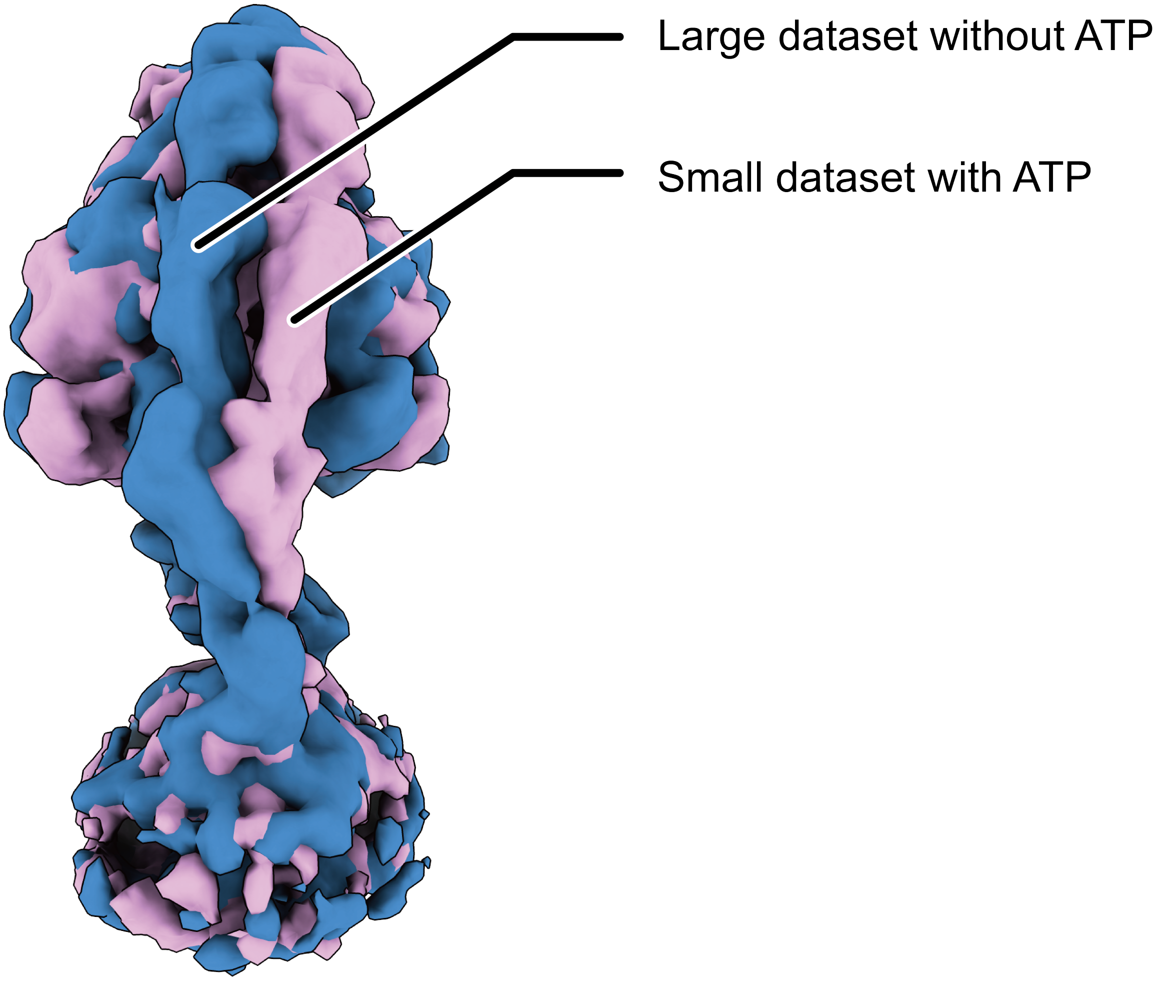

Supplementary Figure 4. Imaging ATP synthase during ATP hydrolysis reveals conformations with a strained peripheral stalk. A small dataset of cryoEM images were collected with a screening microscope from ATP synthase frozen during ATP hydrolysis. Analysis of these images yields 3D classes showing the enzyme with a strained peripheral stalk (*pink*), which are not seen in the absence of free ATP (*blue*).

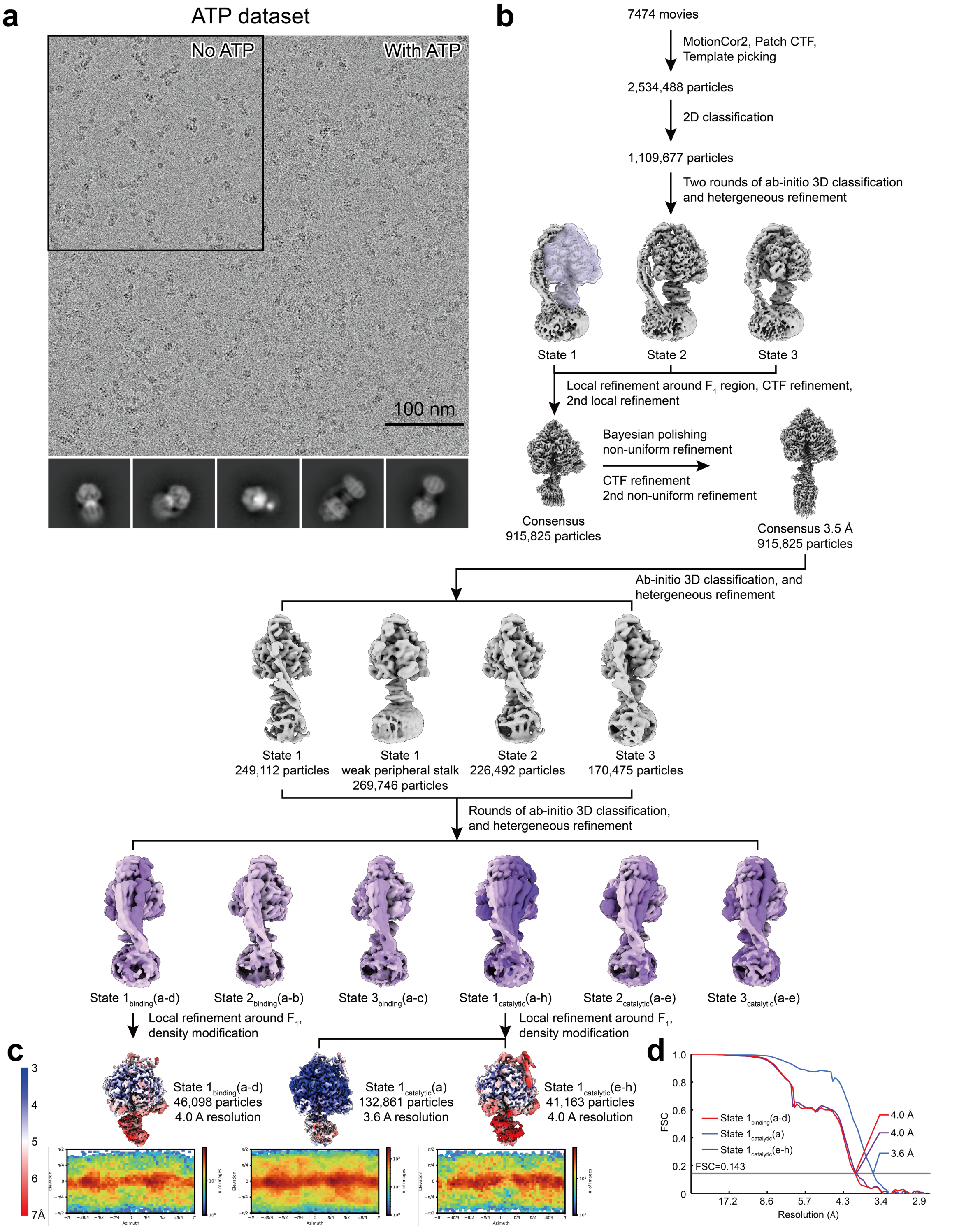

Supplementary Figure 5. CryoEM of the ATP synthase frozen during ATP hydrolysis. A, Example micrograph (1 out of 7474 for ATP dataset and 1 out of 4059 for no ATP dataset) and 2D class average images (5 out of 31). **B,** CryoEM data analysis workflow. **C,** Local resolution maps and orientation distribution plots for the F_1 region in the different conformations. **D,** Fourier shell correlation curves following gold standard refinement and correction of the effects of masking for focused refinement of the F_1 region in the different conformations.

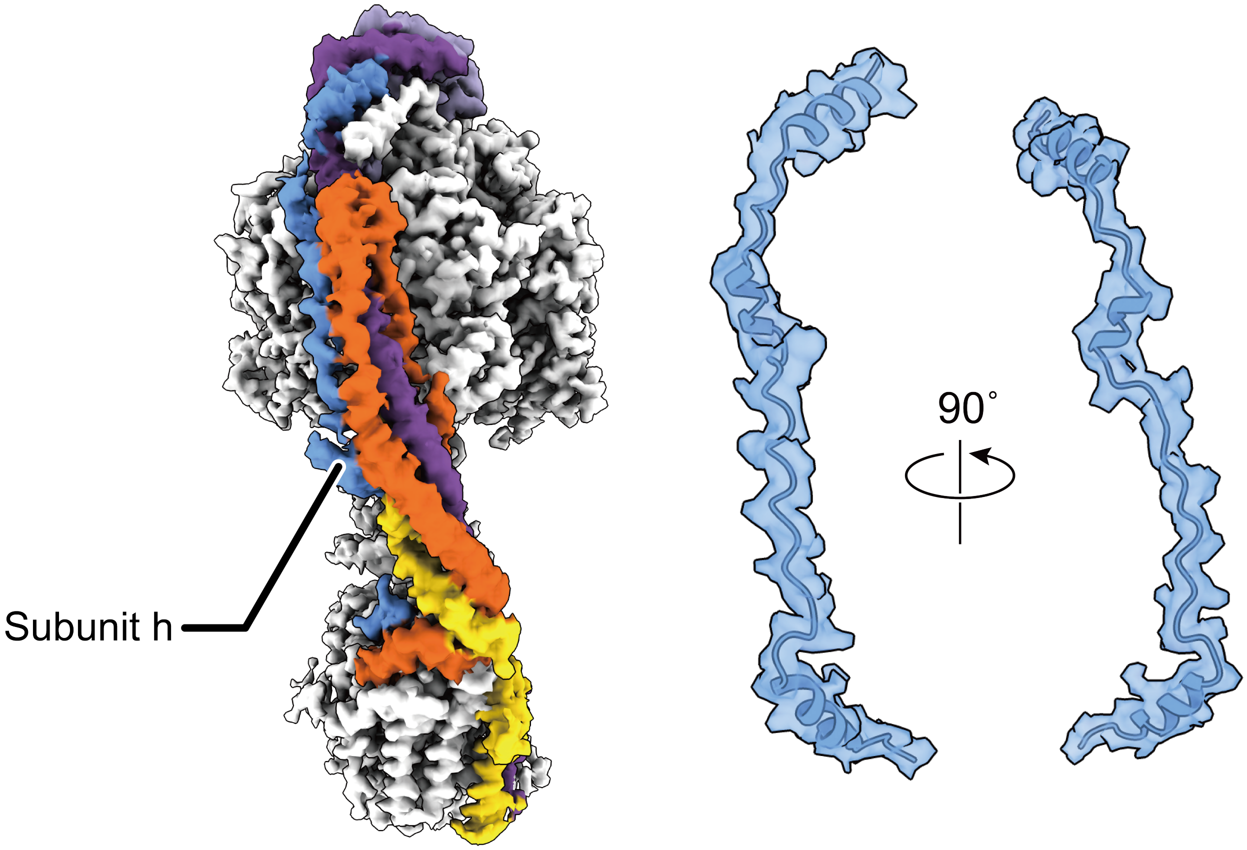

Supplementary Figure 6. Structure of subunit h in the peripheral stalk. Focused refinement of the peripheral stalk revealed nearly-continuous density that accommodates a model of residues 1 to 62 of subunit h from AlphaFold.

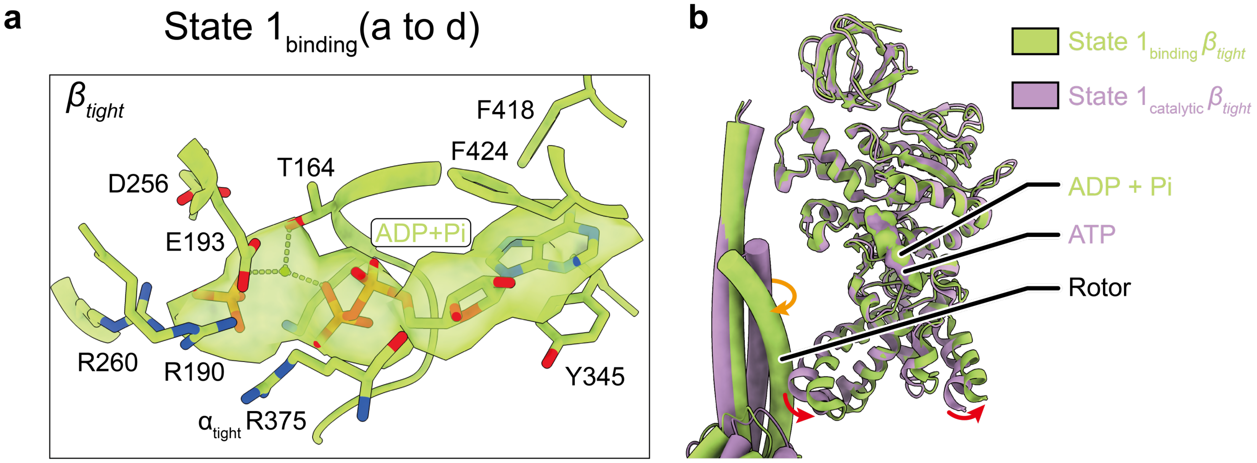

Supplementary Figure 7. Transition from binding to catalytic states is consistent with a previously proposed model of ATP-hydrolysis induced rotation. A, Density for nucleotide in the β_{tight} site of the binding state is consistent with MgADP and Pi. **B,** The binding and catalytic states different by movement of the β subunit (red arrows) and rotation of the γ subunit (orange arrow).

Supplementary Table 1. Cryo-EM data collection, refinement and validation statistics

	ATP free, State 1-3$_{catalytic}$, β_{tight} closed	ATP free, State 1-3$_{catalytic}$, β_{tight} open	ATP free, State 1-3$_{binding}$, β_{tight} open	ATP, State 1$_{catalytic}$(a)	ATP, State 1$_{catalytic}$(e-h)	ATP, State 1$_{binding}$(a-d)
EMDB	25930	25931	25932	25933	25934	25939
PDB	7TJS	7TJT	7TJU	7TJV	7TJW	7TJX
**Data collection and processing**						
Magnification	75,000	75,000	75,000	59,000	59,000	59,000
Voltage (kV)	300	300	300	300	300	300
Electron exposure (e–/Å^2)	39	39	39	40	40	40
Defocus range (μm)	0.5-3.4	0.5-3.4	0.5-3.4	0.5-3.0	0.5-3.0	0.5-3.0
Pixel size (Å)	1.046	1.046	1.046	1.348	1.348	1.348
Symmetry imposed	C1	C1	C1	C1	C1	C1
Initial particle images (no.)	442,025	442,025	442,025	2,534,488	2,534,488	2,534,488
Final particle images (no.)	134,086	144,275	101,012	132,861	41,163	46,098
Map resolution (Å)	3.2	3.2	3.3	3.6	4.0	4.0
FSC threshold	0.143	0.143	0.143	0.143	0.143	0.143
Map resolution range (Å)	2.9-5.5	2.9-5.7	2.9-6.1	3.0-5.6	3.5-7.0	3.5-6.6
**Refinement**						
Initial model used (PDB code)	2HLD	2HLD	2HLD	2HLD	2HLD	2HLD
Model resolution (Å)	3.4	3.4	3.5	3.6	3.8	3.9
FSC threshold	0.5	0.5	0.5	0.5	0.5	0.5
Map sharpening *B* factor (Å^2)	-126.0	-129.5	-123.3	-152.1	-144.0	-151.3
Model composition						
Non-hydrogen atoms	23546	23136	22696	23178	22508	21671
Protein residues	3102	3099	3102	3110	3097	3072
Ligands	8	7	7	10	10	11
B factors (Å^2)						
Protein	38.46	51.29	45.73	63.43	70.19	82.99
Ligand	38.79	46.98	41.61	59.46	61.41	75.03
R.m.s. deviations						
Bond lengths (Å)	0.003	0.003	0.002	0.003	0.003	0.003
Bond angles (°)	0.618	0.642	0.600	0.674	0.651	0.632
Validation						
MolProbity score	0.93	1.11	0.96	0.97	1.07	1.03
Clashscore	1.75	3.24	1.98	2.02	2.42	2.44
EMRinger	3.14	3.19	3.19	2.69	2.06	2.15
Poor rotamers (%)	0.00	0.00	0.00	0.00	0.00	0.05
Ramachandran plot						
Favored (%)	98.54	98.83	98.57	98.06	97.79	98.19
Allowed (%)	1.46	1.17	1.43	1.94	2.21	1.81
Disallowed (%)	0.00	0.00	0.00	0.00	0.00	0.00

Supplementary Table 2. Additional maps and backbone models

	PDB	EMDB	Num. particles	Resolution (Å)	Pixel size (Å)	B factor (Å^2)	Details
State $1_{catalytic}$, β_{tight} closed		25935	56,739	3.4	1.308	-114.0	Locally refined F_1 region generated from the ATP-free dataset (Fig. S1and S2).
State $1_{catalytic}$, β_{tight} open		25936	31,559	3.5	″	-107.1	″
State $1_{binding}$, β_{tight} open		25937	19,922	3.7	″	-100.1	″
State $2_{catalytic}$, β_{tight} closed		25938	52,468	3.4	″	-115.1	″
State $2_{catalytic}$, β_{tight} open		25940	47,065	3.5	″	-110.9	″
State $2_{binding}$, β_{tight} open		25941	57,468	3.4	″	-116.2	″
State $3_{catalytic}$, β_{tight} closed		25942	24,879	3.6	″	-104.6	″
State $3_{catalytic}$, β_{tight} open		25943	65,651	3.4	″	-113.4	″
State $3_{binding}$, β_{tight} open		25944	23,622	3.7	″	-99.3	″
State $1_{catalytic}$(a)	7TJY	25946	41,506	3.8	1.348	Unsharpened	Substate of State $1_{catalytic}$ generated from the ATP-free dataset. Particle number corresponds to particle images used to calculate the map, not the summed number of particle images in this state (Fig. 1F and S1).
State $1_{catalytic}$(b)	7TJZ	25947	8,810	4.4	″	″	″
State $1_{catalytic}$(c)	7TK0	25948	10,550	4.4	″	″	″
State $1_{catalytic}$(d)	7TK1	25949	1,984	7.1	″	″	″
OSCP		25945	191,939	3.3	1.308	-128.6	Locally refined map with particle images from the most relaxed conformations of all three rotational states in the ATP-free dataset (Fig. S1and S2).
Peripheral stalk		25950	191,939	3.7	″	-166.3	″
F_O		25951	191,939	3.7	″	-173.0	″
Composite map		25952			″		Composite map of the ATP-free dataset generated with the locally refined maps of the OSCP, peripheral stalk, F_O, and the State $1_{catalytic}$, β_{tight} closed (Fig. S1and S2).
Map from screening microscope		25953	5,919	8.4	1.348		Map displaying strained peripheral stalk generated with a small dataset collected on the local screening F20 microscope (Fig. S4).
State $1_{binding}$(a)	7TK2	25954	9,369	6.5	″	Unsharpened	Substate generated from the ATP dataset. Particle number corresponds to particle images used to calculate the map, not the summed number of particle images in this state (Fig. 1F and S5).
State $1_{binding}$(b)	7TK3	25955	8,184	6.3	″	″	″
State $1_{binding}$(c)	7TK4	25956	6,644	7.0	″	″	″
State $1_{binding}$(d)	7TK5	25957	3,473	7.8	″	″	″
State $1_{catalytic}$(a)	7TK6	25958	26,797	6.5	″	″	″
State $1_{catalytic}$(b)	7TK7	25959	7,581	6.7	″	″	″
State $1_{catalytic}$(c)	7TK8	25960	16,658	4.7	″	″	″
State $1_{catalytic}$(d)	7TK9	25961	7,185	6.0	″	″	″
State $1_{catalytic}$(e)	7TKA	25962	9,849	7.1	″	″	″
State $1_{catalytic}$(f)	7TKB	25963	6,516	6.3	″	″	″
State $1_{catalytic}$(g)	7TKC	25964	8,727	5.8	″	″	″
State $1_{catalytic}$(h)	7TKD	25965	3,251	7.7	″	″	″
State $2_{binding}$(a)	7TKE	25966	14,051	7.1	″	″	″
State $2_{binding}$(b)	7TKF	25967	9,157	7.1	″	″	″
State $2_{catalytic}$(a)	7TKG	25968	32,285	4.5	″	″	″
State $2_{catalytic}$(b)	7TKH	25969	25,401	4.4	″	″	″
State $2_{catalytic}$(c)	7TKI	25970	4,213	7.1	″	″	″
State $2_{catalytic}$(d)	7TKJ	25971	3,208	7.5	″	″	″
State $2_{catalytic}$(e)	7TKK	25972	3,761	7.3	″	″	″
State $3_{binding}$(a)	7TKL	25973	12,214	6.4	″	″	″
State $3_{binding}$(b)	7TKM	25974	21,305	4.5	″	″	″

State $3_{binding}$(c)	7TKN	25975	16,301	7.1	″	″	″
State $3_{catalytic}$(a)	7TKO	25976	11,541	4.8	″	″	″
State $3_{catalytic}$(b)	7TKP	25977	16,774	4.6	″	″	″
State $3_{catalytic}$(c)	7TKQ	25978	24,660	4.5	″	″	″
State $3_{catalytic}$(d)	7TKR	25979	10,914	6.5	″	″	″
State $3_{catalytic}$(e)	7TKS	25980	3,435	7.5	″	″	″
