# Supplementary material for: Structure of ATP synthase under strain during catalysis
Source: Nat Commun. 2022 Apr 25;13:2232. doi: 10.1038/s41467-022-29893-2 (PMC9038767; doi:10.1038/s41467-022-29893-2)
Supplement: Supplementary file 3 — Description of Additional Supplementary Files [file 41467_2022_29893_MOESM3_ESM.pdf]

Description of Additional Supplementary Files

File Name: Supplementary Movie 1
Description: **Bending of the peripheral stalk in the absence of free ATP and during ATP hydrolysis.** Peripheral stalk bending is illustrated for State $1_{catalytic}$.

File Name: Supplementary Movie 2
Description: **Rotary catalysis cycles for ATP hydrolysis and ATP synthesis.** The rotary cycles are inferred from the least strained conformations observed in the absence of free ATP and compared with the most strained conformations observed during ATP hydrolysis.
