# Supplementary material for: Structure of ATP synthase under strain during catalysis
Source: Nat Commun. 2022 Apr 25;13:2232. doi: 10.1038/s41467-022-29893-2 (PMC9038767; doi:10.1038/s41467-022-29893-2)
Supplement: Supplementary file 6 — Reporting Summary [file 41467_2022_29893_MOESM6_ESM.pdf]

Corresponding author(s): John L. Rubinstein

Last updated by author(s): Jan 26, 2022

Reporting Summary

Nature Portfolio wishes to improve the reproducibility of the work that we publish. This form provides structure for consistency and transparency in reporting. For further information on Nature Portfolio policies, see our Editorial Policies and the Editorial Policy Checklist.

Statistics

For all statistical analyses, confirm that the following items are present in the figure legend, table legend, main text, or Methods section.

n/a	Confirmed	
☒	☐	The exact sample size (*n*) for each experimental group/condition, given as a discrete number and unit of measurement
☒	☐	A statement on whether measurements were taken from distinct samples or whether the same sample was measured repeatedly
☒	☐	The statistical test(s) used AND whether they are one- or two-sided<br>*Only common tests should be described solely by name; describe more complex techniques in the Methods section.*
☒	☐	A description of all covariates tested
☒	☐	A description of any assumptions or corrections, such as tests of normality and adjustment for multiple comparisons
☒	☐	A full description of the statistical parameters including central tendency (e.g. means) or other basic estimates (e.g. regression coefficient) AND variation (e.g. standard deviation) or associated estimates of uncertainty (e.g. confidence intervals)
☒	☐	For null hypothesis testing, the test statistic (e.g. *F*, *t*, *r*) with confidence intervals, effect sizes, degrees of freedom and *P* value noted<br>*Give P values as exact values whenever suitable.*
☒	☐	For Bayesian analysis, information on the choice of priors and Markov chain Monte Carlo settings
☒	☐	For hierarchical and complex designs, identification of the appropriate level for tests and full reporting of outcomes
☒	☐	Estimates of effect sizes (e.g. Cohen's *d*, Pearson's *r*), indicating how they were calculated

Our web collection on statistics for biologists contains articles on many of the points above.

Software and code

Policy information about availability of computer code

Data collection	EPU 2.13
Data analysis	cryoSPARC 3.1-3.3, Topaz 0.2.3, Relion 3.0.8, pyem 0.5, MotionCor 2.1.4.0, Phenix 1.19.2-4158, UCSF Chimera 1.16, UCSF ChimeraX 1.2.5, Isolde 1.2.2, coot 0.9.2, EMRinger 1.0.0, Molprobity 4.5.1

For manuscripts utilizing custom algorithms or software that are central to the research but not yet described in published literature, software must be made available to editors and reviewers. We strongly encourage code deposition in a community repository (e.g. GitHub). See the Nature Portfolio guidelines for submitting code & software for further information.

Data

Policy information about availability of data

All manuscripts must include a data availability statement. This statement should provide the following information, where applicable:

- Accession codes, unique identifiers, or web links for publicly available datasets
- A description of any restrictions on data availability
- For clinical datasets or third party data, please ensure that the statement adheres to our policy

CryoEM maps generated in this study have been deposited in the Electron Microscopy Data Bank with accession numbers 29530 (https://www.ebi.ac.uk/emdb/entry/EMD-29530), 29531 (https://www.ebi.ac.uk/emdb/entry/EMD-29531), 29532 (https://www.ebi.ac.uk/emdb/entry/EMD-29532), 29533 (https://www.ebi.ac.uk/emdb/entry/EMD-29533), 29534 (https://www.ebi.ac.uk/emdb/entry/EMD-29534), 29535 (https://www.ebi.ac.uk/emdb/entry/EMD-29535), 29536 (https://www.ebi.ac.uk/emdb/entry/EMD-29536), 29537 (https://www.ebi.ac.uk/emdb/entry/EMD-29537), 29538 (https://www.ebi.ac.uk/emdb/entry/EMD-29538), 29539 (https://www.ebi.ac.uk/emdb/entry/EMD-29539), 29540 (https://www.ebi.ac.uk/emdb/entry/EMD-29540), 29541 (https://www.ebi.ac.uk/emdb/entry/EMD-29541), 29542 (https://www.ebi.ac.uk/emdb/entry/EMD-29542), 29543 (https://www.ebi.ac.uk/emdb/entry/EMD-29543), 29544 (https://

www.ebi.ac.uk/emdb/entry/EMD-29544), 29545 (https://www.ebi.ac.uk/emdb/entry/EMD-29545), 29546 (https://www.ebi.ac.uk/emdb/entry/EMD-29546), 29547 (https://www.ebi.ac.uk/emdb/entry/EMD-29547), 29548 (https://www.ebi.ac.uk/emdb/entry/EMD-29548), 29549 (https://www.ebi.ac.uk/emdb/entry/EMD-29549), 29550 (https://www.ebi.ac.uk/emdb/entry/EMD-29550), 29551 (https://www.ebi.ac.uk/emdb/entry/EMD-29551), 29552 (https://www.ebi.ac.uk/emdb/entry/EMD-29552), 29553 (https://www.ebi.ac.uk/emdb/entry/EMD-29553), 29554 (https://www.ebi.ac.uk/emdb/entry/EMD-29554), 29555 (https://www.ebi.ac.uk/emdb/entry/EMD-29555), 29556 (https://www.ebi.ac.uk/emdb/entry/EMD-29556), 29557 (https://www.ebi.ac.uk/emdb/entry/EMD-29557), 29558 (https://www.ebi.ac.uk/emdb/entry/EMD-29558), 29559 (https://www.ebi.ac.uk/emdb/entry/EMD-29559), 29560 (https://www.ebi.ac.uk/emdb/entry/EMD-29560), 29561 (https://www.ebi.ac.uk/emdb/entry/EMD-29561), 29562 (https://www.ebi.ac.uk/emdb/entry/EMD-29562), 29563 (https://www.ebi.ac.uk/emdb/entry/EMD-29563), 29564 (https://www.ebi.ac.uk/emdb/entry/EMD-29564), 29565 (https://www.ebi.ac.uk/emdb/entry/EMD-29565), 29566 (https://www.ebi.ac.uk/emdb/entry/EMD-29566), 29567 (https://www.ebi.ac.uk/emdb/entry/EMD-29567), 29568 (https://www.ebi.ac.uk/emdb/entry/EMD-29568), 29569 (https://www.ebi.ac.uk/emdb/entry/EMD-29569), 29570 (https://www.ebi.ac.uk/emdb/entry/EMD-29570), 29571 (https://www.ebi.ac.uk/emdb/entry/EMD-29571), 29572 (https://www.ebi.ac.uk/emdb/entry/EMD-29572), 29573 (https://www.ebi.ac.uk/emdb/entry/EMD-29573), 29574 (https://www.ebi.ac.uk/emdb/entry/EMD-29574), 29575 (https://www.ebi.ac.uk/emdb/entry/EMD-29575), 29576 (https://www.ebi.ac.uk/emdb/entry/EMD-29576), 29577 (https://www.ebi.ac.uk/emdb/entry/EMD-29577), 29578 (https://www.ebi.ac.uk/emdb/entry/EMD-29578), 29579 (https://www.ebi.ac.uk/emdb/entry/EMD-29579), 29580 (https://www.ebi.ac.uk/emdb/entry/EMD-29580), and atomic models have been deposited in the Protein Data Bank with accession codes 7TJS (http://doi.org/10.2210/pdb7TJS/pdb), 7TJT (http://doi.org/10.2210/pdb7TJT/pdb), 7TJU (http://doi.org/10.2210/pdb7TJU/pdb), 7TJV (http://doi.org/10.2210/pdb7TJV/pdb), 7TJW (http://doi.org/10.2210/pdb7TJW/pdb), 7TJX (http://doi.org/10.2210/pdb7TJX/pdb), 7TJY (http://doi.org/10.2210/pdb7TJY/pdb), 7TJZ (http://doi.org/10.2210/pdb7TJZ/pdb), 7TK0 (http://doi.org/10.2210/pdb7TK0/pdb), 7TK1 (http://doi.org/10.2210/pdb7TK1/pdb), 7TK2 (http://doi.org/10.2210/pdb7TK2/pdb), 7TK3 (http://doi.org/10.2210/pdb7TK3/pdb), 7TK4 (http://doi.org/10.2210/pdb7TK4/pdb), 7TK5 (http://doi.org/10.2210/pdb7TK5/pdb), 7TK6 (http://doi.org/10.2210/pdb7TK6/pdb), 7TK7 (http://doi.org/10.2210/pdb7TK7/pdb), 7TK8 (http://doi.org/10.2210/pdb7TK8/pdb), 7TK9 (http://doi.org/10.2210/pdb7TK9/pdb), 7TKA (http://doi.org/10.2210/pdb7TKA/pdb), 7TKB (http://doi.org/10.2210/pdb7TKB/pdb), 7TKC (http://doi.org/10.2210/pdb7TKC/pdb), 7TKD (http://doi.org/10.2210/pdb7TKD/pdb), 7TKE (http://doi.org/10.2210/pdb7TKE/pdb), 7TKF (http://doi.org/10.2210/pdb7TKF/pdb), 7TKG (http://doi.org/10.2210/pdb7TKG/pdb), 7TKH (http://doi.org/10.2210/pdb7TKH/pdb), 7TKI (http://doi.org/10.2210/pdb7TKI/pdb), 7TKJ (http://doi.org/10.2210/pdb7TKJ/pdb), 7TKK (http://doi.org/10.2210/pdb7TKK/pdb), 7TKL (http://doi.org/10.2210/pdb7TKL/pdb), 7TKM (http://doi.org/10.2210/pdb7TKM/pdb), 7TKN (http://doi.org/10.2210/pdb7TKN/pdb), 7TKO (http://doi.org/10.2210/pdb7TKO/pdb), 7TKP (http://doi.org/10.2210/pdb7TKP/pdb), 7TKQ (http://doi.org/10.2210/pdb7TKQ/pdb), 7TKR (http://doi.org/10.2210/pdb7TKR/pdb), 7TKS (http://doi.org/10.2210/pdb7TKS/pdb). Previously published atomic models used for atomic model building are available in the Protein Data Bank with accession codes 2HLD (http://doi.org/10.2210/pdb2HLD/pdb), 6B2Z (http://doi.org/10.2210/pdb6B2Z/pdb), 6CP3 (http://doi.org/10.2210/pdb6CP3/pdb).

Field-specific reporting

Please select the one below that is the best fit for your research. If you are not sure, read the appropriate sections before making your selection.

☒ Life sciences ☐ Behavioural & social sciences ☐ Ecological, evolutionary & environmental sciences

For a reference copy of the document with all sections, see nature.com/documents/nr-reporting-summary-flat.pdf

Life sciences study design

All studies must disclose on these points even when the disclosure is negative.

Sample size	The number of micrographs (4059, 7474) and particle images (442025 and 2,534488) were chosen based on past experience on ATP synthase structure determination so that structures can be determined to sufficient resolution to uncover mechanisms discussed in this work.
Data exclusions	Data rejection was performed by automatic 2D and 3D classification, with class members not corresponding to ATP synthase rejected visually, as it standard in the cryoEM field.
Replication	Reproducibility was ensured by first detecting conformational changes in small test datasets and then collecting the much larger datasets used for high resolution structure determination described in the manuscript. Each dataset has hundreds of thousands to millions of particle images so the method has inherent replication. The larger datasets were not replicated due to high cost of microscope, which is typical in the field.
Randomization	For resolution assessment randomization is by division into odd-even groups
Blinding	Blinding is not possible but is not needed considering the automated handling of large cryoEM image datasets

Reporting for specific materials, systems and methods

We require information from authors about some types of materials, experimental systems and methods used in many studies. Here, indicate whether each material, system or method listed is relevant to your study. If you are not sure if a list item applies to your research, read the appropriate section before selecting a response.

Materials & experimental systems

n/a	Involved in the study
☒	☐ Antibodies
☒	☐ Eukaryotic cell lines
☒	☐ Palaeontology and archaeology
☒	☐ Animals and other organisms
☒	☐ Human research participants
☒	☐ Clinical data
☒	☐ Dual use research of concern

Methods

n/a	Involved in the study
☒	☐ ChIP-seq
☒	☐ Flow cytometry
☒	☐ MRI-based neuroimaging
